# Supplementary material for: Effectiveness of D-dimer in predicting distant metastasis in colorectal cancer
Source: PLoS One. 2024 Jul 12;19(7):e0306909. doi: 10.1371/journal.pone.0306909 (PMC11244829; doi:10.1371/journal.pone.0306909)
Supplement: Attachment — Submitted filename: Response to Reviewers.docx [file pone.0306909.s002.docx]

Dear editor,

Thanks for your comments and suggestions. We have revised our manuscript according to the reviewers' comments, as follows:

Journal requirements:

When submitting your revision, we need you to address these additional requirements.

1. Please ensure that your manuscript meets PLOS ONE's style requirements, including those for file naming. The PLOS ONE style templates can be found at
<https://journals.plos.org/plosone/s/file?id=wjVg/PLOSOne_formatting_sample_main_body.pdf> and
<https://journals.plos.org/plosone/s/file?id=ba62/PLOSOne_formatting_sample_title_authors_affiliations.pdf>

**Our response:** We have made changes to the manuscript according to PLOS ONE's style requirements. In line 7, We changed the font size of ‘Abstract’ to 18pt. We also changed the parentheses of the citations to the middle parentheses.

2. In the online submission form, you indicated that [All relevant data are available from the corresponding author.].
All PLOS journals now require all data underlying the findings described in their manuscript to be freely available to other researchers, either 1. In a public repository, 2. Within the manuscript itself, or 3. Uploaded as supplementary information.
This policy applies to all data except where public deposition would breach compliance with the protocol approved by your research ethics board. If your data cannot be made publicly available for ethical or legal reasons (e.g., public availability would compromise patient privacy), please explain your reasons on resubmission and your exemption request will be escalated for approval.

**Our response:** We have added data to the additional information.

3. Please review your reference list to ensure that it is complete and correct. If you have cited papers that have been retracted, please include the rationale for doing so in the manuscript text, or remove these references and replace them with relevant current references. Any changes to the reference list should be mentioned in the rebuttal letter that accompanies your revised manuscript. If you need to cite a retracted article, indicate the article’s retracted status in the References list and also include a citation and full reference for the retraction notice.
**Our response:** After careful examination, we did not cite the retracted papers.
Additional Editor Comments:

Only minor revisions are required.


[Note: HTML markup is below. Please do not edit.]

Reviewers' comments:

Reviewer's Responses to Questions

**Comments to the Author**

1. Is the manuscript technically sound, and do the data support the conclusions?

The manuscript must describe a technically sound piece of scientific research with data that supports the conclusions. Experiments must have been conducted rigorously, with appropriate controls, replication, and sample sizes. The conclusions must be drawn appropriately based on the data presented.

Reviewer #1: Yes

2. Has the statistical analysis been performed appropriately and rigorously?

Reviewer #1: I Don't Know

3. Have the authors made all data underlying the findings in their manuscript fully available?

The [PLOS Data policy](http://www.plosone.org/static/policies.action#sharing) requires authors to make all data underlying the findings described in their manuscript fully available without restriction, with rare exception (please refer to the Data Availability Statement in the manuscript PDF file). The data should be provided as part of the manuscript or its supporting information, or deposited to a public repository. For example, in addition to summary statistics, the data points behind means, medians and variance measures should be available. If there are restrictions on publicly sharing data—e.g. participant privacy or use of data from a third party—those must be specified.

Reviewer #1: No

4. Is the manuscript presented in an intelligible fashion and written in standard English?

PLOS ONE does not copyedit accepted manuscripts, so the language in submitted articles must be clear, correct, and unambiguous. Any typographical or grammatical errors should be corrected at revision, so please note any specific errors here.

Reviewer #1: Yes

5. Review Comments to the Author

Please use the space provided to explain your answers to the questions above. You may also include additional comments for the author, including concerns about dual publication, research ethics, or publication ethics. (Please upload your review as an attachment if it exceeds 20,000 characters)

Reviewer #1: - Can you explain the fact that sensibility and specificity values of PT, PTA, PTR and INR are so different? They are different ways of the same test, and we do not expect such different results.
**Our response:** Dear reviewer, thank you for your valuable comments. As you said, PT, PTA and PTR are different ways of the same test. It is not difficult to find that they have a similar AUC (95% CI) and Youden Index. Since we determine the cut-off value based on the optimal Youden index, the difference of cut-off value will inevitably cause the difference in sensitivity and specificity, but we can find that their diagnostic efficiency is similar.
- You say: "The levels of PT, PTR, INR, fibrinogen and FDP also elevated in the metastatic group (Table 1, all P<0.05)." I understand that in these tests p-value is between 0.001 and 0.05, but in Table 1, it is marked these p-values are <0.001. Indicate which are the correct p-values.

**Our response:** Dear reviewer, all P values are correct, and the P<0.05 we annotated in the text contains P<0.001.

6. PLOS authors have the option to publish the peer review history of their article ([what does this mean?](https://journals.plos.org/plosone/s/editorial-and-peer-review-process#loc-peer-review-history)). If published, this will include your full peer review and any attached files.


If you choose “no”, your identity will remain anonymous but your review may still be made public.


**Do you want your identity to be public for this peer review?** For information about this choice, including consent withdrawal, please see our [Privacy Policy](https://www.plos.org/privacy-policy).

Reviewer #1: No
